# Increased risk of death from pneumonia among cancer survivors: A propensity score‐matched cohort analysis

**DOI:** 10.1002/cam4.5456

**Published:** 2022-11-21

**Authors:** Shiori Tanaka, Manami Inoue, Taiki Yamaji, Motoki Iwasaki, Tetsuji Minami, Shoichiro Tsugane, Norie Sawada, the JPHC Study Group

**Affiliations:** ^1^ Epidemiology and Prevention Group Institute for Cancer Control, National Cancer Center Tokyo Japan; ^2^ National Insitute of Health and Nutrition National Institutes of Biomedical Innovation, Health and Nutrition Tokyo Japan

**Keywords:** cancer survivors, epidemiology, mortality, pneumonia, propensity score, prospective cohort study

## Abstract

**Background:**

The repeated global pandemic of the new virus has led to interest in the possibility of severe pneumonia among cancer patients and survivors. Here, we aimed to assess the association between incident cancer and risk of death from pneumonia in Japanese in a large population‐based cohort study.

**Methods:**

We used the data from The Japan Public Health Center‐based Prospective Study (JPHC Study), which enrolled subjects aged 40 to 69 between 1990 and 1994 and followed their cancer incidence and mortality until 2013. After identifying 103,757 eligible subjects for analysis and imputing missing data on covariates by the chained equations approach, we conducted propensity score‐matched analysis for 1:4 matching, leaving 14,520 cases diagnosed with cancer and 48,947 controls without cancer during the study period for final analysis. A Cox proportional hazards regression model was used to estimate the hazard ratio (HR) and corresponding confidence interval (CI) for the risk of death from pneumonia with comparison of cancer cases and cancer‐free controls.

**Results:**

Compared to cancer‐free individuals, risk of death from pneumonia was significantly higher among those who had any diagnosed cancer (HR, 1.41; 95%CI, 1.08–1.84); those within 1 year of diagnosis (HR, 23.0; 95% CI, 2.98–177.3); within 1 to <2 years (HR, 3.66; 95% CI, 1.04–12.9); and those with regional spread or distant metastatic cancer at initial diagnosis (HR, 2.01; 95% CI, 1.26–3.21). A history of lung, oesophageal, and head and neck cancer conferred the higher risk among site‐specific cancers.

**Conclusion:**

We found a positive association between incident cancer and risk of death from pneumonia in this study. These results imply the possibility that the immunocompromised status and respiratory failure due to antitumor treatment may have resulted in a more severe outcome from pneumonia among cancer survivors than the general population.

## Graphical abstract

This prospective cohort study found a higher risk of death from pneumonia among cancer survivors compared to cancer‐free controls. The increased risk was pronounced for cases within two years after diagnosis and those with advanced cancer at diagnosis.

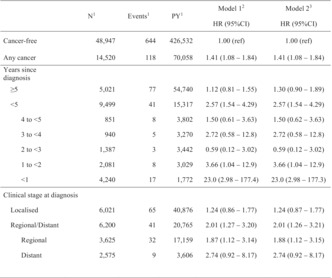

## 1 INTRODUCTION

Globally, pneumonia is the leading cause of death from infection, accounting for 2.5 million deaths in 2019.^1^ Although the disease burden of pneumonia is primarily considered to affect older people worldwide and children under age 5 in developing countries, the repeated emergence of new viruses over the last few decades has re‐emphasised pneumonia as a public health risk.^2^ In Japan, approximately 95,000 deaths were due to pneumonia in 2019, accounting for 6.9% of total deaths, and of which 97% were in patients over 65 years old.^3^

Interest is growing in the risk of viral pneumonia among cancer survivors.^4^, ^5^, ^6^ Cancer patients are more susceptible to infection than the general population because various cancer treatments place them in an immunocompromised state.^7^, ^8^ Previous studies have reported a higher risk of hospitalisation and mortality from pneumonia among patients with haematological malignancies.^9^, ^10^ Patients who undergo treatment for haematological malignancies are more susceptible to infection due to a high degree of defects involving the innate and adaptive immune systems.^11^ Surgical procedures for lung, oesophageal and head and neck (HN) cancers are highly invasive and can have several severe postoperative complications, including pneumonia.^12^ Furthermore, cancer patients often suffer from conditions partially caused by antitumour treatment and various comorbidities, including cardiac disease, diabetes, dyslipidaemia, hypertension and obesity, which may lead to severe pneumonia.^9^, ^11^, ^13^ The risk factors for various cancers are closely similar to those for pneumonia prognosis and other chronic diseases, including older age, smoking, poor diet, obesity and alcohol intake.^14^ Cancer diagnosis and antitumor treatment may be possible risk factors for severe pneumonia,^15^, ^16^, ^17^, ^18^, ^19^ although evidence for this putative association is scarce. Given the above background, the risk of death from pneumonia might be high for cancer survivors, especially lung, oesophageal and HN cancers and haematological malignancies.

Here, we investigated the association between incident cancer and risk of death from pneumonia using a large‐scale, population‐based longitudinal survey in Japan.

## 2 METHODS

### 2.1 Study setting

The study used data from the Japan Public Health Centre‐based Prospective Study (JPHC Study). The JPHC Study consisted of two cohorts: Cohort I (baseline; launched in 1990) and Cohort II (in 1993). In all, 140,420 participants in 11 public health centre areas (PHCs) were enrolled. Participant age ranged from 40–59 years in Cohort I and 40–69 years in Cohort II. Details of the JPHC Study have been described elsewhere.^20^, ^21^

### 2.2 Study population

**FIGURE 1:**
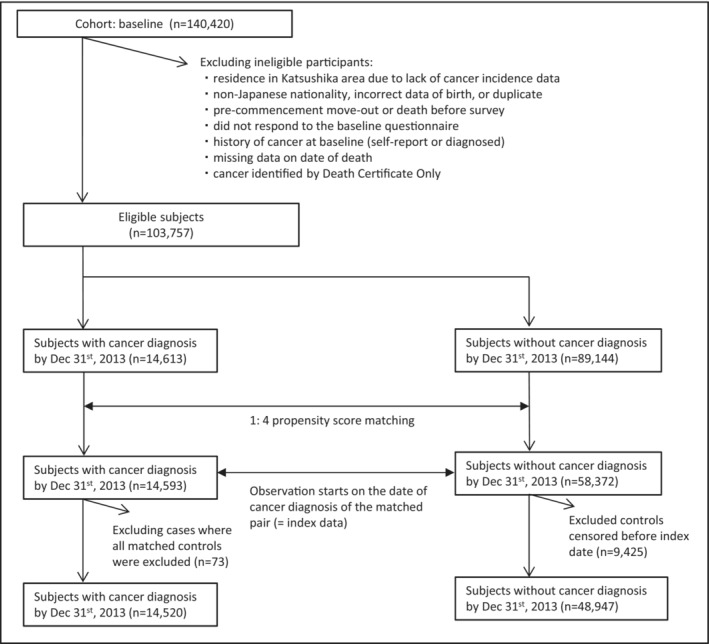
Study flow

Figure 1 shows the participants included in the present study. Participants residing in the Tokyo area were excluded because of the incomplete availability of data on incident cancer. The other exclusion criteria were non‐Japanese nationality, incorrect birth data, multiple registrations, pre‐commencement loss or death, no response to the baseline survey, history of cancer before the baseline survey identified by self‐report or from registries, cancers identified on death certificates only and missing date of death. Finally, the analysis included 103,757 participants. Incorrect birth data was identified through a registration system administered by the local government. In our cancer registry system, very few cancer cases were ascertained only by death certificates (1.2%) and were thus excluded.

The study was approved by the Institutional Review Board of the National Cancer Centre of Japan (approval number: 2001–021) in accordance with the relevant ethics guidelines for medical research in Japan. When each participant completed the baseline questionnaire, the study's purpose and follow‐up methods were fully explained, and informed consent was implicitly obtained.

### 2.3 Assessment of exposure

The baseline survey was conducted from 1990 to 1992 for Cohort I and from 1993 to 1994 for Cohort II. We used any incident cancer that occurred between the baseline survey and 31 December 2013 (or 30 December 2012 for the Suita area). Cancer cases were identified through active patient notifications from major local hospitals and linkages with population‐based cancer registries. Cancer diagnoses were coded using *The International Classification of Diseases for Oncology*, Third Edition. Discrete periods after cancer diagnoses and clinical stages at diagnoses were also used. Based on the Surveillance, Epidemiology, and End Results (SEER) classification, clinical stage at diagnosis was categorised as localised, regional and distant. We used the following site‐specific cancers: lung (C34), oesophagus (C15), head and neck (HN) (C00–C14, C32) and hematologic malignancies (morphology codes 959–972, 974–975, 973–976 and 980–994, respectively) according to previous studies as described in the Introduction section. In cases where a participant was diagnosed with multiple cancers during follow‐up, we used only the first incidence case.

### 2.4 Ascertainment of outcome

Death certificates and permission to identify the cause of death for deceased participants were provided by the Ministry of Health, Labour and Welfare. Causes of death were collected from the primary cause of deaths and classified based on the *International Classification of Diseases, 10th Revision* (*ICD‐10*). Pneumonia death was defined as ICD‐10 codes J10‐J18. Information on the incidence of pneumonia was not available in this study.

### 2.5 Statistical analysis

We conducted an analysis based on propensity scores in accordance with the methodology of Saito et al.^22^ First, we conducted multiple imputation by chained equations with 20 iterations to address missing data under the assumption that they were missing at random. The covariates included into the propensity score analysis were age at baseline (40–44, 45–49, 50–54, 55–59, 60–64, or 65+ years), PHC area (10 PHCs), sex, smoking status (never, former, or current), alcohol intake (never/former or <1 time/week), regular (ethanol converted g/week [1 to <150, 150 to <300, or ≥ 300]), body mass index (BMI) (in kg/m^2^ [< 18.5, 18.5–<25, 25–<30, or ≥ 30]), weekly leisure‐time sports or physical exercise (<1 time, 1–4 time(s), or ≥5 times), history of second‐hand smoke at home (no or yes), history of second‐hand smoke in the workplace (never, sometimes, or always), coffee intake (almost never, <1 cup/d, or ≥1 cup/d), green tea intake (almost never, <1 cup/d, or ≥1 cup/d), and history of diabetes (no or yes). Person‐years, history of asthma, and vital status were used as auxiliary variables. Second, we conducted logistic regression using the covariates described above to estimate the probability of developing cancer based on the baseline variables in each imputed dataset.^23^ Then, we used a pooled propensity score to match, with replacement, subjects with and without incident cancer in a 1:4 ratio. We established a calliper width of 0.25 of the propensity score's standard deviation (SD), resulting in a better balance of subjects with incident cancer and those without as matched samples (C‐statistic = 0.67). We assessed the distributions of basic characteristics among groups with standardised mean differences (SMD).

Third, we defined an index date for cases as the date of cancer diagnosis. Accordingly, cancer‐free controls were assigned the index date corresponding to the date of cancer diagnosis of their matched cases. All subjects were followed until the date of death, migration out of the study area or end of follow‐up (31 December 2013), whichever occurred first. We excluded controls who were censored before the index date. If all four matched controls were excluded, their matched case was also excluded. Finally, Cox proportional hazards regression models using attained age as the time scale were employed to estimate the hazard ratio (HR) of mortality from pneumonia with comparison of cancer cases to controls stratified by the propensity score‐matched pairs (Model 1). A history of asthma at the baseline survey was further adjusted for as a risk factor for pneumonia (no or yes) (Model 2). We also analysed the association between any incident cancer and pneumonia death using years after cancer diagnosis (cancer‐free, 5+ years, 4–<5 years, 3–4 years, 2–<3 years, 1–2 years, or 0–1 years) and clinical stage at cancer diagnosis (localised, regional or distant).

We further conducted the same approach for each cancer site as secondary analyses. Considering the possibility of misclassifying a death from cancer as from pneumonia and vice versa,^24^ we also analysed the models following exclusion of cases diagnosed within 1 year after diagnosis as a sensitivity analysis. All *p* values were two‐sided and had values smaller than 0.05, indicating statistical significance. All analyses were conducted using STATA version 16.0 software (StataCorp LP).

## 3 RESULTS

The propensity scores matching analysis included 103,757 subjects from the JPHC Study during a mean follow‐up time of 17.8 years. The mean age at study entry (±standard deviation) was 53.0 (±7.8) years, and the proportion of female was 52.3%. After matching, controls who were censored before the index date (*n* = 9425) and those who lost all matched controls (*n* = 73) were excluded, leaving 14,520 cases and 48,947 controls as final analytic samples. Table 1 shows the baseline characteristics of the included participants with and without incident cancer during follow‐up. Comparison of SMD, age, sex, study area, smoking status and alcohol intake improved the balance between cases and controls after matching. During 496,590 person‐years of follow‐up, we identified 762 deaths from pneumonia after 1:4 propensity score matching with replacement. The number of site‐specific cancers was 1885 for lung, 411 for oesophagus, 426 for HN, 747 for haematologic and 11,088 for other cancers.

**TABLE 1:** Basic characteristics of study subjects by propensity score‐matched pair at baseline survey in the JPHC study

Variable	Category	Unmatched^a^			Matched^a^		
		Cancer (*n* = 14,613)	Cancer‐free (*n* = 89,144)	SMD	Cancer (*n* = 14,520)	Cancer‐free (*n* = 48,947)	SMD
Age	Mean (SD)	54.7 (7.7)	51.3 (8.0)	−0.43	54.7 (7.7)	54.3 (7.6)	−0.05
Sex	Female, %	39.2	55.7	0.33	38.4	41.4	0.06
Study area	Iwate, %	8.1	9.1	0.20	8.1	8.3	0.08
BMI	Mean (SD)	23.5 (3.0)	23.6 (3.3)	−0.04	23.5 (3.1)	23.6 (3.3)	−0.03
Smoking	Current, %	38.6	27.1	0.31	38.4	37.4	0.04
Alcohol	Non‐drinker, %	46.1	52.9	0.22	45.6	47.4	0.04
Leisure‐time exercise	<1 times/w, %	81.7	81.4	0.05	81.7	83.9	0.06
Passive smoking at home	Yes, %	65.0	66.4	0.03	65.0	66.3	0.03
Passive smoking at workplace	Every day, %	36.3	33.4	0.08	36.2	36.5	0.04
Coffee intake	≥1 cup/d, %	35.9	41.2	0.11	35.5	35.6	0.02
Green tea intake	≥1 cup/d, %	77.3	74.4	0.07	77.3	79.6	0.06
History of diabetes	Yes, %	5.9	4.4	0.07	5.9	4.6	0.06

Abbreviations: SD, standard deviation; SMD, standardised mean difference.

^a^ Estimate using multiple imputation by chained equations with 20 iterations, including age, public health centre area, gender, smoking, status, alcohol intake, body mass index, weekly leisure‐time sports or physical exercise, history of second‐hand smoke at home, history of second‐hand smoke in the workplace, coffee intake, green tea intake, and history of diabetes and person‐years, history of asthma, and vital status used as auxiliary variables.

Table 2 presents the HR with corresponding 95% confidence intervals (CI) for the association between incident cancer, years since diagnosis and clinical stage at diagnosis for any cancer and the risk of death from pneumonia. Compared to cancer‐free individuals, those who had any diagnosed cancer (HR, 1.41; 95% CI, 1.08–1.84), those within 1 year of diagnosis (HR, 23.0; 95% CI, 2.98–177.3) or within 1 to <2 years (HR, 3.66; 95% CI, 1.04–12.9) and those whose cancer was regionally spread or distantly metastatic at diagnosis (HR, 2.01; 95%CI, 1.26–3.21) all had a significantly higher risk of dying from pneumonia. In contrast, more than 2 years after diagnosis, risk in those with cancers that were localised at diagnosis was no different from that of cancer‐free individuals.

**TABLE 2:** Hazard ratios (HR) and 95% confidence intervals (CIs) of death from pneumonia during follow‐up by incident cancer in the JPHC study

	*N* ^a^	Events^a^	PY^a^	Model 1^b^	Model 2^c^
				HR (95%CI)	HR (95%CI)
Cancer‐free	48,947	644	426,532	1.00 (ref)	1.00 (ref)
Any cancer	14,520	118	70,058	1.41 (1.08–1.84)	1.41 (1.08–1.84)
Years since diagnosis					
≥5	5021	77	54,740	1.12 (0.81–1.55)	1.30 (0.90–1.89)
<5	9499	41	15,317	2.57 (1.54–4.29)	2.57 (1.54–4.29)
4 to <5	851	8	3802	1.50 (0.61–3.63)	1.50 (0.62–3.63)
3 to <4	940	5	3270	2.72 (0.58–12.8)	2.72 (0.58–12.8)
2 to <3	1387	3	3442	0.59 (0.12–3.02)	0.59 (0.12–3.02)
1 to <2	2081	8	3029	3.66 (1.04–12.9)	3.66 (1.04–12.9)
<1	4240	17	1772	23.0 (2.98–177.4)	23.0 (2.98–177.3)
Clinical stage at diagnosis					
Localised	6021	65	40,876	1.24 (0.86–1.77)	1.24 (0.87–1.77)
Regional/distant	6200	41	20,765	2.01 (1.27–3.20)	2.01 (1.26–3.21)
Regional	3625	32	17,159	1.87 (1.12–3.14)	1.88 (1.12–3.15)
Distant	2575	9	3606	2.74 (0.92–8.17)	2.74 (0.92–8.17)

Abbreviations: CI, confidence interval; HR, hazard ratio; N, number; PY, person‐year.

^a^ The number was calculated after matching by pooled propensity score for subjects with and without incident cancer during follow‐up in a 1:4 ratio with replacement, in which propensity scores were estimated by age at baseline (40–44, 45–49, 50–54, 55–59, 60–64, or 65+ years old), public health centre area (10 areas), sex, smoking status (never, former, or current), history of second‐hand smoke at home (no or yes), history of second‐hand smoke at the workplace (never, sometimes, or always), alcohol intake (never/former, <1 time/week), regular (ethanol converted g/day) [<23, 23–<46, 46–<69, 69–<92, or ≥92], BMI (in kg/m^2^; <18.5, 18.5–<25, 25–<30, or ≥30), weekly leisure‐time sports or physical exercise (<1 time, 1–4 time(s), or ≥5 times), coffee intake (almost never, <1 cup/d, or ≥1 cup/d), green tea intake  (almost never, <1 cup/d, or ≥1 cup/d), and history of diabetes (no or yes).

^b^ Cox proportional hazards model using attained age as time scale stratified on the propensity‐score matched pairs.

^c^ Model 2 adjusted for history of asthma (no or yes).

Table 3 shows the association between the incidence of site‐specific cancers and risk of dying from pneumonia. Compared to cancer‐free controls, those with a history of lung cancer, lung cancer diagnosed less than 5 years before and lung cancer with regional spread or distant metastasis at diagnosis were significantly associated with a higher risk of death from pneumonia. Similarly, a history of oesophageal or HN cancer, oesophageal cancer diagnosed more than 5 years before, localised oesophageal cancer at diagnosis, and HN cancer with regional spread or distant metastasis were associated with an increased risk of death from pneumonia. No associations were found for a history of haematological malignancy or other cancers. Among cases with other cancers, the increased risk was found only in those with a diagnosis within the last 5 years. Risk was diminished for the model which excluded cases within 1 year of diagnosis, but was significantly higher among those with regional spread or distant metastases (Table 4).

**TABLE 3:** Hazard ratios (HR) and 95% confidence intervals (CIs) of death from mortality during follow‐up by specific cancers in the JPHC study

		*N* ^a^	Events^a^	PY^a^	Model 1^b^	Model 2^c^
					HR (95%CI)	HR (95%CI)
Cancer‐free		6175	116	51,874	1.00 (ref)	1.00 (ref)
Lung cancer		1885	16	5404	3.34 (1.27–8.80)	3.42 (1.28–9.16)
Years since diagnosis	≥5	361	6	3581	2.06 (0.57–7.39)	2.10 (0.57–7.75)
	<5	1524	10	1822	6.53 (1.29–33.0)	6.53 (1.29–32.9)
Clinical stage at diagnosis	Localised	533	5	3111	5.37 (1.003–28.7)	6.04 (1.02–35.9)
	Regional/ Distant	1157	9	1885	2.11 (0.60–7.52)	2.12 (0.60–7.52)
Cancer‐free		1364	20	12,316	1.00 (ref)	1.00 (ref)
Oesophageal cancer		411	8	1422	4.38 (1.25–15.3)	4.38 (1.25–15.3)
Years since diagnosis	≥5	98	5	973	5.83 (1.10–30.8)	5.83 (1.10–30.8)
	<5	313	3	449	2.83 (0.39–20.7)	2.83 (0.39–20.9)
Clinical stage at diagnosis	Localised	135	5	843	6.29 (1.21–32.8)	6.29 (1.21–32.8)
	Regional/Distant	226	2	474	2.40 (0.31–18.4)	2.40 (0.31–18.4)
Cancer‐free		1422	18	12,478	1.00 (ref)	1.00 (ref)
Head and neck cancer		426	7	2135	4.31 (1.19–15.6)	3.91 (1.07–14.3)
Years since diagnosis	≥5	138	5	1623	4.11 (0.91–18.5)	3.58 (0.78–16.5)
	<5	283	2	501	4.89 (0.41–58.4)	4.89 (0.41–58.4)
Clinical stage at diagnosis	Localised	153	0	1076	–	–
	Regional/Distant	204	6	739	16.0 (1.90–135.2)	14.2 (1.67–120.9)
Cancer‐free		2573	26	22,719	1.00 (ref)	1.00 (ref)
Haematological malignancies		747	3	2661	0.83 (0.21–3.23)	0.83 (0.23–3.23)
Years since diagnosis	≥5	177	3	1826	1.10 (0.26–4.60)	1.10 (0.26–4.60)
	<5	570	0	835	–	–
Cancer‐free		37,462	436	329,167	1.00 (ref)	1.00 (ref)
Other cancers		11,088	86	58,728	1.20 (0.88–1.64)	1.18 (0.86–1.62)
Years since diagnosis	≥5	4259	59	46,966	1.00 (0.69–1.45)	0.98 (0.67–1.42)
	<5	6829	27	11,762	2.07 (1.10–3.90)	2.08 (1.10–3.93)
Clinical stage at diagnosis	Localised	5137	56	35,537	1.30 (0.88–1.93)	1.28 (0.86–1.90)
	Regional/distant	4427	24	17,113	1.41 (0.79–2.50)	1.38 (0.77–2.46)

Abbreviations: CI, confidence interval; HR, hazard ratio; N, number; PY, person‐year.

^a^ The number was calculated after matching by pooled propensity score for subjects with and without incident cancer during follow‐up in a 1:4 ratio with replacement, in which propensity scores were estimated by age at baseline (40–44, 45–49, 50–54, 55–59, 60–64, or 65+ years old), public health centre area (10 areas), sex, smoking status (never, former, or current), history of second‐hand smoke at home (no or yes), history of second‐hand smoke at the workplace (never, sometimes, or always), alcohol intake (never/former, <1 time/week), regular (ethanol converted g/day) [<23, 23–<46, 46–<69, 69–<92, or ≥92], BMI (in kg/m^2^; <18.5, 18.5–<25, 25–<30, or ≥30), weekly leisure‐time sports or physical exercise (<1 time, 1–4 time(s), or ≥5 times), coffee intake (almost never, <1 cup/d, or ≥1 cup/d), green tea intake (almost never, <1 cup/d, or ≥1 cup/d), and history of diabetes (no or yes).

^b^ Cox proportional hazards model using attained age as time scale stratified on the propensity‐score matched pairs.

^c^ Model 2 adjusted for history of asthma (no or yes).

**TABLE 4:** Hazard ratios (HR) and 95% confidence intervals (CIs) of death from mortality during follow‐up by any incident cancer excluding those with a follow‐up time less than 1 year since diagnosis in the JPHC study

	*N* ^a^	Events^a^	PY^a^	Model 1^b^	Model 2^c^
				HR (95%CI)	HR (95%CI)
Cancer‐free	35,022	444	319,880	1.00 (ref)	1.00 (ref)
Any cancer	10,280	101	68,285	1.24 (0.93–1.61)	1.24 (0.93–1.64)
Years since diagnosis					
≥5	5021	77	54,740	1.12 (0.81–1.55)	0.99 (0.70–1.41)
1 to <5	5266	24	13,545	1.73 (0.97–3.10)	1.73 (0.97–3.10)
Clinical stage at diagnosis					
Localised	5385	59	40,564	1.13 (0.78–1.63)	1.12 (0.78–1.63)
Regional/distant	3411	33	19,637	1.75 (1.07–2.86)	1.74 (1.06–2.85)

Abbreviations: CI, confidence interval; HR, hazard ratio; N, number; PY, person‐year.

^a^ The number was calculated after matching by pooled propensity score for subjects with and without incident cancer during follow‐up in a 1:4 ratio with replacement, in which propensity scores were estimated by age at baseline (40–44, 45–49, 50–54, 55–59, 60–64, or 65+ years old), public health centre area (10 areas), sex, smoking status (never, former, or current), history of second‐hand smoke at home (no or yes), history of second‐hand smoke at the workplace (never, sometimes, or always), alcohol intake (never/former, <1 time/week), regular (ethanol converted g/day) [<23, 23–<46, 46–<69, 69–<92, or ≥92], BMI (in kg/m^2^; <18.5, 18.5–<25, 25–<30, or ≥30), weekly leisure‐time sports or physical exercise (<1 time, 1–4 time(s), or ≥5 times), coffee intake (almost never, <1 cup/d, or ≥1 cup/d), green tea intake (almost never, <1 cup/d, or ≥1 cup/d), and history of diabetes (no or yes).

^b^ Cox proportional hazards model using attained age as time scale stratified on the propensity‐score matched pairs.

^c^ Model 2 adjusted for history of asthma (no, or yes).

## 4 DISCUSSION

This study found a positive association between incident cancer and higher risk of death from pneumonia compared to cancer‐free controls in the Japanese population, including variations by cancer site, time since diagnosis and clinical stage at diagnosis. In particular, risk was higher in those less than 2 years from diagnosis and those whose cancers were regionally spread or distantly metastasised at diagnosis. Secondary analyses for site‐specific cancers revealed that patients with lung, oesophageal, and HN cancers had a higher risk of dying from pneumonia than cancer‐free controls. In contrast, no associations were found with haematological malignancies and other cancers. To our knowledge, this is the first time a large‐scale prospective cohort study has been used in a general population in Japan to quantify the relative risk of death from pneumonia in cancer survivors.

Although a systematic review found that incident cancer was not a definitive risk factor for pneumonia,^14^ an excess risk of pneumonia deaths among cancer patients has been consistently reported in recent years. Previous reports have shown that cancer patients were 2 to 21 times more likely to die from pneumonia than the general population.^15^, ^16^, ^17^ These results had greater impact than our estimate because they focused on deaths during the short term. Helena et al. reported higher risks of hospitalisation and mortality from influenza among those who survived cancer for more than 1 year than among cancer‐free controls.^18^ Risk diminished with time since diagnosis, and no difference was observed at 10 years after diagnosis.^18^

Surveys of childhood cancer survivors have reported that antitumour treatment causes late pulmonary toxicity and chronic pulmonary complications, limits daily activity, increases oxygen need and causes recurrent pneumonia and subsequent excess pneumonia death.^19^ Those who survive cancer may be affected by acute, iatrogenic or treatment‐induced infections.^25^ Both the cancer itself and various antitumour treatments can impair functional status and drastically alter the host's immune system, placing them in an immunosuppressed condition.^19^ Immune system suppression using corticosteroids and other immunosuppressive agents or bone marrow suppression caused by cytotoxic agents render patients more prone to infection.^8^ Direct irradiation of the chest and lungs can cause treatment‐induced pneumonitis and interstitial lung injury.^26^ Postoperative pneumonia, although not a focus of this study, is one of the most common complications in patients with lung, oesophagus and HN cancers undergoing surgical resection.^27^ The occurrence of postoperative pneumonia can lead to prolonged hospital stays and respiratory failure.^28^ Advanced cancer weakens the patient's immune system and requires multimodality therapies, resulting in immunocompromise and increasing the risk of infectious disease.^29^, ^30^ Increased exposure to resistant bacteria during frequent treatment may lead to treatment failure and severe pneumonia.^31^ In the present study, the increased risk of pneumonia within one or 2 year(s) after cancer diagnosis may be reflective of cases under active treatment, as the entire course of some anticancer regimens extends over more than a year.^32^, ^33^ Higher risk of death from pneumonia among cancer survivors may also be explained by the high overlap between the risk factors for developing cancer and for pneumonia death (e.g., tobacco exposure).^10^, ^34^ Other risk factors for pneumonia death may also include common cancer comorbidities or/and comorbidities subsequent to antitumour treatment (e.g., cardiac disease, diabetes, dyslipidaemia, hypertension and obesity).^9^, ^11^, ^13^

The high risk of pneumonia among lung cancer patients is consistently attributable to bronchial obstructions caused by tumours, bronchoscopy, surgery and coexisting structural lung diseases.^15^ In particular, advanced lung cancer is an independent risk factor associated with pneumonia, mainly as a result of the long‐term anatomical abnormalities caused by pneumonectomy.^35^, ^36^ This study showed an increased risk in localised lung cancer but not advanced cases, likely because of a high rate of cancer death in advanced lung cancer cases. Surgery for oesophageal cancer frequently results in postoperative complications, which may be one reason for the low pneumonia survival rates among those with this cancer type.^37^ HN cancer patients commonly experience a long‐term dysphagia, various adverse respiratory outcomes and pneumonia after curative surgery and chemoradiotherapy.^38^, ^39^, ^40^ In the present study, regional or distant metastases of HN cancer were associated with pneumonia death. Pulmonary metastases frequently occur in HN cancers and require pulmonary metastasectomies,^41^ which may explain the higher pneumonia risk among patients with advanced HN cancer. These findings suggest that a diagnosis of cancer, especially cancer of the lung, oesophagus and HN, should receive higher attention in efforts aimed at preventing pneumonia.

Although some studies have shown a higher risk of pneumonia among haematological malignancies, others have reached inconsistent conclusions.^8^, ^42^ Data from the present study showed no similar associations, possibly because the classification of haematological malignancies differed among some studies and was not determined in others.

For other types of cancer, the present study found no association between incident cancer and death from pneumonia. The mix of various cancer types might have diluted the association between incident cancer and pneumonia deaths in other types of cancer. Analysis for the most common types of cancers, including stomach, colon, liver, pancreas, prostate, and breast cancer showed null associations. Given that gastric and liver cancers are mostly caused by infectious agents, and breast and prostate cancers are often caused by sex hormones, we speculate that people with these cancers were less affected by risk factors of pneumonia deaths than those with lung, HN and oesophagus cancers, resulting in the lack of association with pneumonia deaths. The association was not prominent even in non‐aggressive cancers, which might be ascribable to less competition from primary cancer deaths, such as prostate cancer. This null association may be partially because cancer survivors appear more likely than the general population to receive vaccination against pneumococcal and influenza viruses due to guideline recommendations.^43^ Lifestyle improvements and increased opportunities to access healthcare after diagnosis may also guard against infection.^11^ Primary cancer deaths might compete with pneumonia deaths.^25^ Competing risk of death from primary cancer may explain the null association for aggressive cancers such as pancreas cancer. Nevertheless, we decided that competing risk assessment was not appropriate in an association study,^44^ as an analysis accounting for competing risk may underestimate the relative risk or result in protective pneumonia deaths, leading to erroneous interpretation.

### 4.1 Strengths and limitations

The study has several noteworthy advantages, including its large sample size, high response rate (81%) and low loss to follow‐up. Our analysis using propensity score matching provides robust evidence to mitigate the critical differences in risk factors between cancer cases and controls. Detailed information on the date of cancer diagnosis, clinical stage and site‐specific cancers enabled us to assess the gradient of risk among cancer survivors. The study's subjects reflected Japan's general population, making our findings applicable to the nation as a whole.

This study has some limitations. First, because we had no detailed data on cancer treatments, we could not analyse by treatment type or status (whether patients were under active treatment or not). Different regimens may have different effects on pneumonia outcomes.^15^, ^45^ Therefore, the inability to assess the impact of cancer treatment may have led to overestimating or underestimating results. Second, although the prospective nature of our study enabled robust adjustment of confounders, we could not rule out the possibility of residual confounding. The study did not obtain data on pneumococcal vaccination, which may have a positive effect on pneumonia outcomes. Without adjustment for vaccination history, our study may have underestimated the effects. Third, we could not assess the impact of lifestyle change or the status of comorbidities after a cancer diagnosis. Fourth, our exclusion of subjects living in Tokyo, an urban area, could have resulted in selection bias because the different lifestyle and better access to medical care of urban compared to rural areas may influence cancer incidence and pneumonia death. Finally, physicians may have made errors in coding the cause of death because of competing effects in comorbidity‐associated mortality,^24^ especially in cases where the patient was under active treatment based on an expert's opinion. We addressed this issue by excluding those diagnosed within 1 year as a treatment period. We further confirmed that the rate of pneumonia death among cancer cases did not substantially differ by study area, indicating that cause of death was accurately or at least consistently identified nationwide.

Allowing for these limitations, our study nevertheless provides insightful evidence regarding pneumonia in cancer survivors. As mechanisms of tissue injury in pneumonia induced by viral agents seem to share some aspects with severe acute respiratory syndrome coronavirus 2 (SARV‐CoV‐2),^46^ it is intriguing to speculate that our results are extrapolatable to coronavirus disease 2019 (COVID‐19)‐induced pneumonia, which is caused by SARS‐CoV‐2. Further study of the association between cancer history and COVID‐19 outcomes in the current global pandemic is warranted.

## 5 CONCLUSIONS

This prospective cohort study found a higher risk of death from pneumonia among cancer survivors compared to cancer‐free controls. The increased risk was pronounced for cases within 2 years after diagnosis and those with advanced cancer at diagnosis. By site, lung, oesophageal, and HN cancer survivors had a higher risk of death from pneumonia. The results may suggest that the possible immunocompromised status and respiratory failure of patients following antitumor treatment impacted the greater severity of outcomes from pneumonia in cancer survivors than in the general population.^47^ These vulnerable people may need to be given special precautions to prevent infection, including optimization of vaccination and patient education.

## AUTHOR CONTRIBUTIONS

**Shiori Tanaka:** Conceptualization (lead); formal analysis (lead); methodology (lead); visualization (lead); writing – original draft (lead); writing – review and editing (lead). **Manami Inoue:** Conceptualization (supporting); supervision (lead); writing – original draft (supporting); writing – review and editing (supporting). **Taiki Yamaji:** Writing – review and editing (supporting). **Motoki Iwasaki:** Writing – review and editing (supporting). **Tetsuji Minami:** Conceptualization (equal); data curation (equal); writing – review and editing (equal). **Shoichiro Tsugane:** Funding acquisition (lead); project administration (lead); writing – review and editing (supporting). **Norie Sawada:** Funding acquisition (lead); investigation (lead); project administration (lead); writing – review and editing (supporting).

## FUNDING INFORMATION

This work was funded by the National Cancer Centre Research and Development Fund (since 2010); and a Grant‐in‐Aid for Cancer Research from the Ministry of Health, Labour and Welfare of Japan.

## CONFLICT OF INTEREST

The authors declare no conflicts of interest.

## Data Availability

The datasets analysed during the current study are not available due to the denial of permission from the ethical board of our institution. For information on how to apply to gain access to JPHC data and/or biospecimens, please follow the instructions at https://epi.ncc.go.jp/en/jphc/805/8155.html.

## References

[1] SOCIETY AT . Urgent Progress needed to end the preventable burden of pneumonia and deaths: the forum of international respiratory societies. Accessed 30 September, 2021. https://www.thoracic.org/about/newsroom/press‐releases/journal/2020/urgent‐progress‐needed‐to‐end‐the‐preventable‐burden‐of‐pneumonia‐and‐deaths‐firs.php

[2] Greenslade L . World pneumonia day during a global pneumonia pandemic: 12 November 2020. Am J Physiol Lung Cell Mol Physiol. 2020;319(5):L859‐L860. doi:10.1152/ajplung.00462.2020 32997505 PMC7839243

[3] Ministry of Health, Labour and Welfare . Vital Statistics Annual Report 2019. Accessed Aug 10 2022. https://www.mhlw.go.jp/toukei/saikin/hw/jinkou/geppo/nengai19/dl/gaikyouR1.pdf

[4] Watson M , Fielding R , Lam W , Pirl W . COVID‐19, cancer and psycho‐oncology: dealing with the challenges. Psychooncology. 2020;29(9):1373. doi:10.1002/pon.5467 33448491

[5] Kim YJ , Lee ES , Lee YS . High mortality from viral pneumonia in patients with cancer. Infect Dis (Lond). 2019;51(7):502‐509. doi:10.1080/23744235.2019.1592217 31081422

[6] Russell B , Moss C , George G , et al. Associations between immune‐suppressive and stimulating drugs and novel COVID‐19‐a systematic review of current evidence. Ecancermedicalscience. 2020;14:1022. doi:10.3332/ecancer.2020.1022 32256705 PMC7105343

[7] Zhang H , Han H , He T , et al. Clinical characteristics and outcomes of COVID‐19‐infected cancer patients: a systematic review and meta‐analysis. J Natl Cancer Inst. 2021;113(4):371‐380. doi:10.1093/jnci/djaa168 33136163 PMC7665647

[8] Hijano DR , Maron G , Hayden RT . Respiratory viral infections in patients with cancer or undergoing hematopoietic cell transplant. Front Microbiol. 2018;9:3097. doi:10.3389/fmicb.2018.03097 30619176 PMC6299032

[9] Edgington A , Morgan MA . Looking beyond recurrence: comorbidities in cancer survivors. Clin J Oncol Nurs. 2011;15(1):E3‐E12. doi:10.1188/11.Cjon.E3-e12 21278033

[10] Sarfati D , Koczwara B , Jackson C . The impact of comorbidity on cancer and its treatment. CA Cancer J Clin. 2016;66(4):337‐350. doi:10.3322/caac.21342 26891458

[11] Han HJ , Nwagwu C , Anyim O , Ekweremadu C , Kim S . COVID‐19 and cancer: from basic mechanisms to vaccine development using nanotechnology. Int Immunopharmacol. 2021;90:107247. doi:10.1016/j.intimp.2020.107247 33307513 PMC7709613

[12] Andalib A , Ramana‐Kumar AV , Bartlett G , Franco EL , Ferri LE . Influence of postoperative infectious complications on long‐term survival of lung cancer patients: a population‐based cohort study. J Thorac Oncol. 2013;8(5):554‐561. doi:10.1097/JTO.0b013e3182862e7e 23459402

[13] Søgaard M , Thomsen RW , Bossen KS , Sørensen HT , Nørgaard M . The impact of comorbidity on cancer survival: a review. Clin Epidemiol. 2013;5(Suppl 1):3‐29. doi:10.2147/clep.S47150 24227920 PMC3820483

[14] Almirall J , Serra‐Prat M , Bolíbar I , Balasso V . Risk factors for community‐acquired pneumonia in adults: a systematic review of observational studies. Respiration. 2017;94(3):299‐311. doi:10.1159/000479089 28738364

[15] Schmedt N , Heuer OD , Häckl D , Sato R , Theilacker C . Burden of community‐acquired pneumonia, predisposing factors and health‐care related costs in patients with cancer. BMC Health Serv Res. 2019;19(1):30. doi:10.1186/s12913-018-3861-8 30642312 PMC6332528

[16] Pelton SI , Shea KM , Farkouh RA , et al. Rates of pneumonia among children and adults with chronic medical conditions in Germany. BMC Infect Dis. 2015;15(1):470. doi:10.1186/s12879-015-1162-y 26515134 PMC4627378

[17] Kolditz M , Tesch F , Mocke L , Höffken G , Ewig S , Schmitt J . Burden and risk factors of ambulatory or hospitalized CAP: a population based cohort study. Respir Med. 2016;121:32‐38. doi:10.1016/j.rmed.2016.10.015 27888989

[18] Carreira H , Strongman H , Peppa M , et al. Prevalence of COVID‐19‐related risk factors and risk of severe influenza outcomes in cancer survivors: a matched cohort study using linked English electronic health records data. EClinicalMedicine. 2020;29‐30:100656. doi:10.1016/j.eclinm.2020.100656 PMC7788436 33437952

[19] Dietz AC , Chen Y , Yasui Y , et al. Risk and impact of pulmonary complications in survivors of childhood cancer: a report from the childhood cancer survivor study. Cancer. 2016;122(23):3687‐3696. doi:10.1002/cncr.30200 27504874 PMC5115933

[20] Watanabe S , Tsugane S , Sobue T , Konishi M , Baba S . Study design and organization of the JPHC study. Japan public health center‐based prospective study on cancer and cardiovascular diseases. J Epidemiol. 2001;11(6 Suppl):S3‐S7. doi:10.2188/jea.11.6sup_3 11763137 PMC11858366

[21] Tsugane S , Sawada N . The JPHC study: design and some findings on the typical Japanese diet. Jpn J Clin Oncol. 2014;44(9):777‐782. doi:10.1093/jjco/hyu096 25104790

[22] Saito E , Inoue M , Sawada N , et al. Risk of stroke in cancer survivors using a propensity score‐matched cohort analysis. Sci Rep. 2021;11(1):5599. doi:10.1038/s41598-021-83368-w 33692383 PMC7946896

[23] Ling AY , Montez‐Rath ME , Mathur MB , Kapphahn KI , Desai M . How to apply multiple imputation in propensity score matching with partially observed confounders: a simulation study and practical recommendations. arXiv: Applications. 2019.

[24] Mieno MN , Tanaka N , Arai T , et al. Accuracy of death certificates and assessment of factors for misclassification of underlying cause of death. J Epidemiol. 2016;26(4):191‐198. doi:10.2188/jea.JE20150010 26639750 PMC4808686

[25] Zaorsky NG , Churilla TM , Egleston BL , et al. Causes of death among cancer patients. Ann Oncol. 2017;28(2):400‐407. doi:10.1093/annonc/mdw604 27831506 PMC5834100

[26] Arroyo‐Hernández M , Maldonado F , Lozano‐Ruiz F , Muñoz‐Montaño W , Nuñez‐Baez M , Arrieta O . Radiation‐induced lung injury: current evidence. BMC Pulm Med. 2021;21(1):9. doi:10.1186/s12890-020-01376-4 33407290 PMC7788688

[27] Simonsen DF , Søgaard M , Bozi I , Horsburgh CR , Thomsen RW . Risk factors for postoperative pneumonia after lung cancer surgery and impact of pneumonia on survival. Respir Med. 2015;109(10):1340‐1346. doi:10.1016/j.rmed.2015.07.008 26209227

[28] Fernandez‐Bustamante A , Frendl G , Sprung J , et al. Postoperative pulmonary complications, early mortality, and hospital stay following noncardiothoracic surgery: a multicenter study by the perioperative research network investigators. JAMA Surg. 2017;152(2):157‐166. doi:10.1001/jamasurg.2016.4065 27829093 PMC5334462

[29] Perry A , Lee SH , Cotton S , Kennedy C . Therapeutic exercises for affecting post‐treatment swallowing in people treated for advanced‐stage head and neck cancers. Cochrane Database Syst Rev. 2016;2016(8):CD011112. doi:10.1002/14651858.CD011112.pub2 27562477 PMC7104309

[30] Hiam‐Galvez KJ , Allen BM , Spitzer MH . Systemic immunity in cancer. Nat Rev Cancer. 2021;21(6):345‐359. doi:10.1038/s41568-021-00347-z 33837297 PMC8034277

[31] Perdikouri EIA , Arvaniti K , Lathyris D , et al. Infections due to multidrug‐resistant bacteria in oncological patients: insights from a five‐year epidemiological and clinical analysis. Microorganisms. 2019;7(9):277. doi:10.3390/microorganisms7090277 31438593 PMC6780124

[32] Yajima S , Shimizu H , Sakamaki H , Ikeda S , Ikegami N , Murayama J‐I . Real‐world cost analysis of chemotherapy for colorectal cancer in Japan: detailed costs of various regimens during the entire course of chemotherapy. BMC Health Serv Res. 2016;16(1):2. doi:10.1186/s12913-015-1253-x 26728154 PMC4698819

[33] Tsutsué S , Tobinai K , Yi J , Crawford B . Nationwide claims database analysis of treatment patterns, costs and survival of Japanese patients with diffuse large B‐cell lymphoma. PloS One. 2020;15(8):e0237509. doi:10.1371/journal.pone.0237509 32810157 PMC7444590

[34] Bello S , Menéndez R , Antoni T , et al. Tobacco smoking increases the risk for death from pneumococcal pneumonia. Chest. 2014;146(4):1029‐1037. doi:10.1378/chest.13-2853 24811098

[35] Mulder RL , Thönissen NM , van der Pal HJ , et al. Pulmonary function impairment measured by pulmonary function tests in long‐term survivors of childhood cancer. Thorax. 2011;66(12):1065‐1071. doi:10.1136/thoraxjnl-2011-200618 21803931

[36] Huang TT , Hudson MM , Stokes DC , Krasin MJ , Spunt SL , Ness KK . Pulmonary outcomes in survivors of childhood cancer: a systematic review. Chest. 2011;140(4):881‐901. doi:10.1378/chest.10-2133 21415131 PMC3904488

[37] Tamagawa A , Aoyama T , Tamagawa H , et al. Influence of postoperative pneumonia on esophageal cancer survival and recurrence. Anticancer Res. 2019;39(5):2671‐2678. doi:10.21873/anticanres.13392 31092467

[38] Xu B , Boero IJ , Hwang L , et al. Aspiration pneumonia after concurrent chemoradiotherapy for head and neck cancer. Cancer. 2015;121(8):1303‐1311. doi:10.1002/cncr.29207 25537836 PMC4774546

[39] Kawakita D , Abdelaziz S , Chen Y , et al. Adverse respiratory outcomes among head and neck cancer survivors in the Utah cancer survivors study. Cancer. 2020;126(4):879‐885. doi:10.1002/cncr.32617 31721181 PMC6992515

[40] Baxi SS , Pinheiro LC , Patil SM , Pfister DG , Oeffinger KC , Elkin EB . Causes of death in long‐term survivors of head and neck cancer. Cancer. 2014;120(10):1507‐1513. doi:10.1002/cncr.28588 24863390 PMC4101810

[41] Shiono S , Kawamura M , Sato T , et al. Pulmonary metastasectomy for pulmonary metastases of head and neck squamous cell carcinomas. Ann Thorac Surg. 2009;88(3):856‐860. doi:10.1016/j.athoracsur.2009.04.040 19699911

[42] Hermann B , Lehners N , Brodhun M , et al. Influenza virus infections in patients with malignancies––characteristics and outcome of the season 2014/15. A survey conducted by the infectious diseases working party (AGIHO) of the German Society of Haematology and Medical Oncology (DGHO). Eur J Clin Microbiol Infect Dis. 2017;36(3):565‐573. doi:10.1007/s10096-016-2833-3 27838792 PMC5309266

[43] Kawakami K , Nakamura A , Wakana A , Folaranmi TA , Iino T . A Japanese nationwide survey of 23‐valent pneumococcal capsular polysaccharide vaccine (PPSV23) coverage among patients with chronic medical condition aged 50 and older. Hum Vaccin Immunother. 2020;16(7):1521‐1528. doi:10.1080/21645515.2019.1690332 31799889 PMC7482782

[44] Austin PC , Lee DS , Fine JP . Introduction to the analysis of survival data in the presence of competing risks. Circulation. 2016;133(6):601‐609. doi:10.1161/CIRCULATIONAHA.115.017719 26858290 PMC4741409

[45] Su Q , Zhu EC , Wu J‐b , et al. Risk of pneumonitis and pneumonia associated with immune checkpoint inhibitors for solid tumors: a systematic review and meta‐analysis. Front Immunol. 2019;10:108. doi:10.3389/fimmu.2019.00108 30778352 PMC6369169

[46] Miyazawa M . Immunopathogenesis of SARS‐CoV‐2‐induced pneumonia: lessons from influenza virus infection. Inflamm Regen. 2020;40:39. doi:10.1186/s41232-020-00148-1 33062077 PMC7549344

[47] Denlinger CS , Carlson RW , Are M , et al. Survivorship: introduction and definition. Clinical practice guidelines in oncology. J Natl Compr Canc Netw. 2014;12(1):34‐45. doi:10.6004/jnccn.2014.0005 24453291 PMC4465253

